# Isorhamnetin Enhances the Radiosensitivity of A549 Cells Through Interleukin-13 and the NF-κB Signaling Pathway

**DOI:** 10.3389/fphar.2020.610772

**Published:** 2021-01-25

**Authors:** Yarong Du, Cong Jia, Yan Liu, Yehua Li, Jufang Wang, Kun Sun

**Affiliations:** ^1^ College of Life Science, Northwest Normal University, Lanzhou, China; ^2^ Key Laboratory of Space Radiobiology of Gansu Province & CAS Key Laboratory of Heavy Ion Radiation Biology and Medicine, Institute of Modern Physics, Chinese Academy of Sciences, Lanzhou, China; ^3^ School of Nuclear Science and Technology, University of Chinese Academy of Sciences, Beijing, China

**Keywords:** isorhamnetin, IL-13, NF-kB, radiosensitivity, radiotherapy

## Abstract

Isorhamnetin (ISO), a naturally occurring plant flavonoid, is widely used as a phytomedicine. The major treatment modality for non-small-cell lung carcinoma (NSCLC) is radiotherapy. However, radiotherapy can induce radioresistance in cancer cells, thereby resulting in a poor response rate. Our results demonstrated that pretreatment with ISO induced radiosensitizing effect in A549 cells using colony formation, micronucleus, and γH2AX foci assays. In addition, ISO pretreatment significantly enhanced the radiation-induced incidence of apoptosis, the collapse of mitochondrial membrane potential, and the expressions of proteins associated with cellular apoptosis and suppressed the upregulation of NF-κBp65 induced by irradiation in A549 cells. Interestingly, the expression of interleukin-13 (IL-13), an anti-inflammatory cytokine, was positively correlated with the ISO-mediated radiosensitization of A549 cells. The knockdown of IL-13 expression by RNA interference decreased the IL-13 level and thus reduced ISO-mediated radiosensitivity in cells. We also found that the IR-induced NF-κB signaling activation was inhibited by ISO pretreatment, and it was abrogated in IL-13 silenced cells. We speculated that ISO may confer radiosensitivity on A549 cells via increasing the expression of IL-13 and inhibiting the activation of NF-κB. To our knowledge, this is the first report demonstrating the effects of ISO treatment on the responsiveness of lung cancer cells to irradiation through IL-13 and the NF-κB signaling pathway. In summary, ISO is a naturally occurring radiosensitizer with a potential application in adjuvant radiotherapy.

## Introduction

Isorhamnetin (ISO), a flavonoid isolated from traditional Chinese medicine (*Hippophae L.*), has been known to have antioxidative and anti-inflammatory effects and immunomodulatory properties (Echeverry et al., 2004; Seo et al., 2014). In addition, ISO can play a tumor-suppressive role in diverse human tumors as well (Teng et al., 2006; Manu et al., 2015). Zhang et al. reported that ISO could induce mitotic block in non-small-cell lung carcinoma cells (NSCLC), thus enhancing carboplatin- and cisplatin-induced G2/M arrest (Zhang et al., 2015), suggesting that ISO might be a potential clinical chemotherapeutic drug for NSCLC. ISO restrains the proliferation and colony formation and induces the apoptosis of A549 cells, which may be related to mitochondrial dependent pathway. Through disrupting mitochondrial membrane potential (MMP), ISO promoted the release and activation of cytochrome c and caspases 3 and 9 and then induced A549 cells apoptosis (Li et al., 2015).

Lung cancer is one of the most frequently diagnosed cancers in terms of both incidence and mortality, with almost 1.38 million deaths every year worldwide (Alberg et al., 2005; Smith et al., 2019). NSCLC accounts for almost 80% of lung cancers, among which the most common subtypes are lung adenocarcinoma (LUAD) and lung squamous cell carcinoma (LUSC) (Posther and Harpole, 2006; Chen et al., 2017). Radiotherapy is a widely used therapeutic modality for patients in NSCLC. However, NSCLC has been historically considered as a radioresistant malignancy; therefore, conventional chemotherapy or radiotherapy is usually poor in cure rate for cancer patients. More attentions have been attracted to radiosensitizers because of their abilities to increase the radiosensitivity of cancer cells and reduce the side effects on normal cells. In order to identify promising radiosensitivity agents, a large number of natural products with anti-inflammatory, antioxidant, and antitumor activations have been considered (Li et al., 2017; Sun et al., 2017). ISO, a natural product, has these features and is also safe, easily available, and of better efficacy. Can it be potentially served as a radiosensitizer for cancer treatment?

The transcription factor Nuclear Factor-κB (NF-κB) is retained in the cytoplasm in a form of primary heterodimer containing P50 and P65 subunits bound to I-κB. When I-κB is phosphorylated and degraded, NF-κB would be activated, and p50 and p65 translocate to the nucleus and bind to its specific DNA site, which results in a series of key downstream proteins mediating antiapoptosis and attenuates radiation-induced apoptosis (Wang et al., 1996). Several reports have shown that NF-κB activation was associated with worse survival and radioresistance in multiple types of cancers (Magne et al., 2006; Bai et al., 2015; Wu et al., 2015). Thus, the therapeutic effect of radiotherapy is closely related to the active state of NF-κB and the level of inflammatory factor. Inhibition of NF-κB, therefore, has the potential to improve radiotherapeutic efficacy by enhancing radiation-induced cell kill. ISO, as a candidate to inhibit inflammation, has been shown to regulate immune responses via blocking the activation of NF-κB signal and downregulating the secretion of proinflammatory cytokines (TNF-α, IL-1β, and IL-6) (Chi et al., 2016; Li et al., 2016), which may suggest the potential of ISO as a radiosensitizer.

In the present study, we investigated the effects of treatments with ISO alone, irradiation (IR) alone, and combination of ISO + IR on cell viability, DNA damage repair, and apoptosis to determine the radiosensitivity of ISO. Meanwhile, we investigated the mechanisms underlying this effect likely involving NF-κB pathway. Our results demonstrated that ISO could sensitize A549 cells to radiation via inhibition of NF-κB activation mediated by upregulating the level of anti-inflammatory interleukin-13 (IL-13).

## Materials and Methods

### Cell Culture

The non-small-cell lung carcinoma A549 and H460 cells were obtained from Shanghai Cell Bank of Chinese Academic of Science and maintained in RPMI 1640 medium (Gibco, United States) containing 10% heat-inactivated fetal bovine serum (Hyclone, United States) and 1% penicillin/streptomycin at 37°C in a humidified atmosphere containing 95% air and 5% CO_2_.

### Chemical Treatment

ISO was purchased from Baoji Herbest Bio-Tech Company (CAS No: 480-19-3, China; with purity >98%) and stored as 100 mM stock solution in DMSO, protected from light at −20°C.

### Irradiation

Cells were incubated with or without ISO at concentration of 20 μM for 24 h and then irradiated with the indicated dose of X-rays, which were generated by XRad225 (PRECISION X-RAY) operated at 50 kVp energy. The dose rate was about 1.3 Gy/min.

### MTT Assay

Cells were seeded in a 96-well plate at a density of 5 × 10^3^ cells/well and treated with various concentrations of ISO (0, 5, 10, 20, 40, 50, 60, or 80 μM). The control group was treated with an equal volume of DMSO. At the appropriate timepoints, cell proliferation assay was performed by the addition of 50 μl of 3-(4,5-dimethylthiazol-2-yl)-2,5-diphenyltetrazolium bromide (MTT) stock solution (2 mg/ml). After 4 h, the formazan crystals in each well were dissolved by addition of DMSO (150 μl). The absorbance was measured at 550 nm using an automated microplate reader.

### Colony Formation Assay

Cells were pretreated with or without 20 μM ISO for 24 h and then followed by irradiation at the exposure doses of 0, 0.5, 1, 2, 4, or 6 Gy. Cells were irradiated and then immediately reseeded at different densities to yield approximately 50–100 surviving colonies in the ф60 mm petri dish. After being cultured for 12–14 days, the colonies were stained with 0.5% crystal violet for 30 min and manually counted. The survival fraction was generated from three independent experiments with colony numbers normalized to Sham-treated controls.

### Micronucleus Assay

Micronucleus (MN) formation assay is another generally used biological endpoint for potential genotoxic study. Cells were pretreated with or without ISO for 24 h followed by X-rays. After being cultured for 48 h again, cells were fixed with methanol/acetic acid (3:1 v/v) for 20 min at room temperature and then air-dried cells were stained with 20 μl of Acridine Orange in an aqueous solution (10 μg/ml). The analyses were done under the fluorescence microscope (Axio Imager. Z2) at ×20 magnification and the scoring of MN was performed following the criteria established by Fenech (Fenech, 2000). At least 500 cells were scored for each sample. Each experiment was repeated three times independently at least.

### Immunostaining

Immunochemical staining of cells was performed as described (Aten et al., 2004) for DNA double-strand breaks. The cells were reseeded at a density of 1 × 10^5^ cells in 35 mm cell culture dishes overnight and were then pretreated with or without ISO for 24 h followed by X-rays. After irradiation, cells were fixed with 4% paraformaldehyde for 20 min at different time points. The fixed cells were permeabilized with 0.5% Triton X-100 in 10% BSA for 1 h. Subsequent experimental procedures followed the previous description (Jia et al., 2020). The cells were incubated with anti-γH2AX (surrogate of DSB) antibody (1:5,000, Abcam, United States) for 1 h at room temperature, washed three times with PBS, and then incubated in buffer containing the FITC-conjugated goat anti-mouse secondary antibody (1:2,500, ZSGB-BIO, China) for 1 h. After washing with PBS for five times, cells were counterstained with DAPI (Invitrogen, United States). The fluorescent images were taken under a fluorescence microscope (Axio Imager Z2) at ×63 magnification and were analyzed. The fraction of cell with γ-H2AX foci was calculated (numbers of cells with DSBs/total cells) (d’Adda di Fagagna et al., 2003). At least 100 cells were counted for each sample.

### Western Blot Assay

Cells were pretreated with or without 20 μM ISO for 24 h followed by 2 Gy X-rays irradiation. After 12 h, cells were lysed with RIPA buffer (Beyotime, China) with Protease Inhibitor Cocktail Tablets (Roche, Switzerland). Equal amounts of total protein were denatured with 1× loading buffer (Beyotime, China) and subjected to 12% SDS-PAGE and then transferred to a methanol-activated PVDF membrane (Millipore, United States). The membranes were blocked and then probed overnight at 4°C with the following primary antibodies against: Bax, Bcl2, NF-κB, IL-13Rα2, and GAPDH (1:1,000, Proteintech, China); IL-13, IL-13Rα1, (1:1,000, Affinity Biosciences, United States); p-IκBα and p-NF-κB (1:800, CST, United States). After three washes with TBS, membranes were probed with the horseradish peroxidase- (HRP-) labeled secondary antibody (1:2,500, ZSGB-BIO, China) for 1 h at room temperature. Staining was visualized using enhanced chemiluminescence (ECL) reagents, according to the manufacturer’s recommendations. The intensity of protein bands on the western blot image was quantified by Image J software.

### Apoptosis Assays

The cells were incubated with or without 20 μM ISO for 24 h and then irradiated by 2 Gy X-rays. After 48 h, the cells (>1 × 10^5^ cells) were collected, washed, and stained with Annexin V-FITC and propidium iodide (PI) kit (BD, United States) for 15 min according to the manufacturer’s protocol. The ratio of apoptosis was measured by a Coulter Epics on a flow cytometer FlowSight (Amnis, United States).

### Level of Cytokines Measurement by Meso Scale Discovery Assay

The A549 cells were pretreated with or without 20 μM ISO for 24 h and then exposed to 2 Gy X-rays. After 12 h, the supernatant was collected by the centrifuge and detected by MSD assay. According to the manufacturer’s instruction of MSD kit, the MSD V-Plex Proinflammatory Panel 1 Human Kit (MSD platform) (Rockville, MD, United States) was used to measure IL-6, IL-8, IL-10, IL-13, IL-1β, IL-2, IL-4, IFN-γ, IL-12p70, and TNF-α concentrations in cell medium by MSD instrument. Ten kinds of cytokines can be detected simultaneously one-off in each well.

### Mitochondrial Membrane Potential Measurement

JC-1 (BD Biosciences), a dye that can selectively enter into mitochondria and reversibly change color from green to red, was used to detect the MMP levels according to the manufacturer’s instruction. Briefly, after being pretreated with or without ISO for 24 h followed by X-rays irradiation and then being cultured for 24 h again, the cells (∼1 × 10^6^ cells/ml) were loaded in 500 μl JC-1 working solution at 37°C for 20 min and then washed twice with 1× assay buffer. We detected the fluorescence intensity of JC-1 monomers green fluorescence (λex 488 nm, λem 530 nm) as well as JC-1aggregates red fluorescence (λex 525 nm, λem 590 nm). The ratio of fluorescence intensity was calculated to reflect the MMP.

### RNA Interference

IL13 siRNA-1 that targets IL-13 homo-205 (sense: 5′ GCA​GCA​UGG​UAU​GGA​GCA​UTT-3′, antisense: 5′ AUG​CUC​CAU​ACC​AUG​CUG​CTT-3′) and IL13 siRNA-2 that targets IL-13 homo-25 (sense: 5′ UCC​UCA​AUC​CUC​UCC​UGU​UTT-3′, antisense: 5′ AAC​AGG​AGA​GGA​UUG​AGG​ATT-3′) were purchased from GenePharma (Guangzhou, China). A549 cells were transiently transfected with nontargeting siRNA or IL-13 siRNA oligos at a final concentration of 50 nM. The efficacy of interfering IL-13 expression was detected by western blot and quantitative real-time polymerase chain reaction (qRT-PCR). Twenty-four hours after transfection using jetPEI (Polyplus, United States), the cells were treated with or without 20 μM ISO for additional 24 h and then exposed to 2 Gy X-rays. The cells were further subjected to apoptosis detection, MN, and western blotting.

### Cell Growth Curve

The cells were transfected with IL-13 siRNA, pretreated with or without ISO for 24 h, and then exposed to 2 Gy X-rays. The cells were trypsinized soon after irradiation and reseeded in ф60 mm petri dish with 1 × 10^5^ cells/dish for four days and counted by automated cell counter (Z2, Beckman, United States).

### Quantitative Real-Time Polymerase Chain Reaction

According to manufacturer’s instructions, total RNA was isolated using Trizol (Invitrogen). To obtain the cDNA, reverse transcription was conducted using a high-capacity Transcriptor First Strand cDNA synthesis kit (Roche, Switzerland). qRT-PCR was performed with SYBR Green Mix kit (Roche, Switzerland) by using Bio-Rad CFX system. The specific primer for detection of IL-13 gene was F: CAA​TGG​CAG​CAT​GGT​ATG; R: ATC​CTC​TGG​GTC​TTC​TCG. The primer for GAPDH gene was F: GAA​GGT​GAA​GGT​CGG​AGT; R: CAT​GGG​TGG​AAT​CAT​ATT​GGA​A. The mRNA expression levels were normalized to GAPDH using the 2^−ΔΔCt^ method.

### Statistics

The results were presented as means ± standard errors (SE) from at least three independent experiments. The significance of differences (*p* value) was determined by Student’s *t*-test for single comparisons and analysis of variance (ANOVA) for statistical comparison between different groups. The *p* values of 0.05 or less were regarded as significant in two sample’s comparison.

## Results

### Isorhamnetin Treatment Induces Vacuolation and Inhibits Cell Proliferation of Non-Small-Cell Lung Carcinoma Cells

**FIGURE 1:**
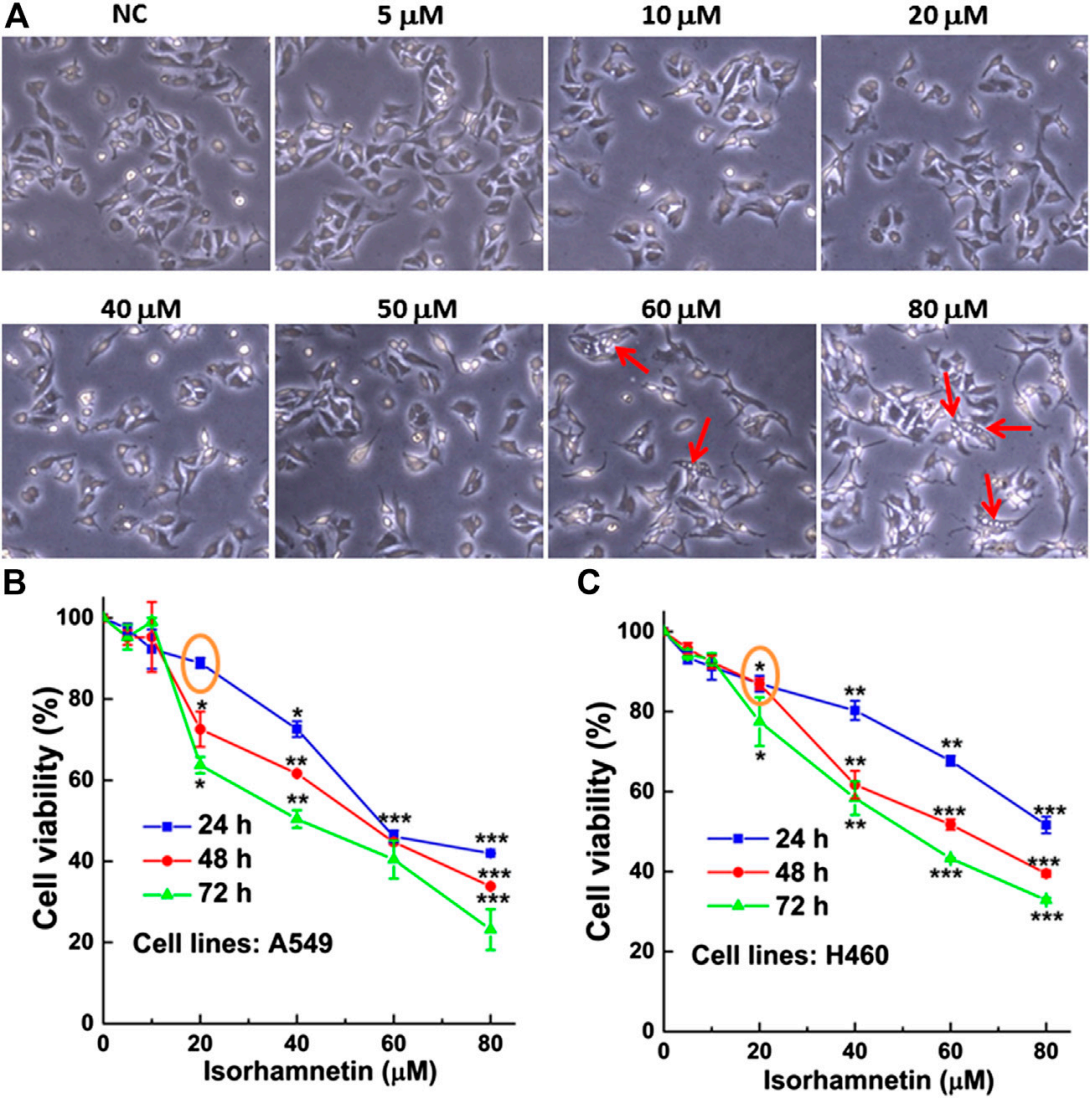
ISO treatment induces vacuolation of NSCLC cells and inhibits cell proliferation. **(A)** The morphology of A549 cells after treatment with different concentrations (0, 5, 10, 20, 40, 50, 60, and 80 μM) of ISO for 24 h. The red arrow indicates the vacuolated A549 cells. The inhibitory effect of ISO was detected by MTT assay after different time of ISO treatment on the proliferations of A549 **(B)** and H460 **(C)** cell lines. *p < 0.05, **p < 0.01, ***p < 0.001 vs. the control groups.

After treatment with ISO (5, 10, 20, 40, 60, and 80 μM) for 24 h, the morphology of A549 cells was altered, and cells were round. Cell vacuolation and disintegration were observed in a dose-dependent manner (Figure 1A). The results of the MTT assay showed that the viability of ISO-treated A549 and H460 cells decreased in concentration- and time-dependent manners. The viability of both A549 (Figure 1B) and H460 (Figure 1C) cells was ∼50% that of respective controls after treatment with 60 μM ISO for 24 h and >85% that of respective controls after treatment with 20 μM ISO, indicating that ISO inhibited cell proliferation.

### Isorhamnetin Enhances the Radiosensitivity of A549 Cells

To investigate whether ISO treatment could enhance the radiosensitivity of cells, two NSCLC cell lines were treated with 20 μM ISO for 24 h and then irradiated with different doses of radiation. Colony formation, micronucleus, and γH2AX foci (a surrogate marker for DNA damage) assay were performed to examine the degree of radiosensitivity.

**FIGURE 2:**
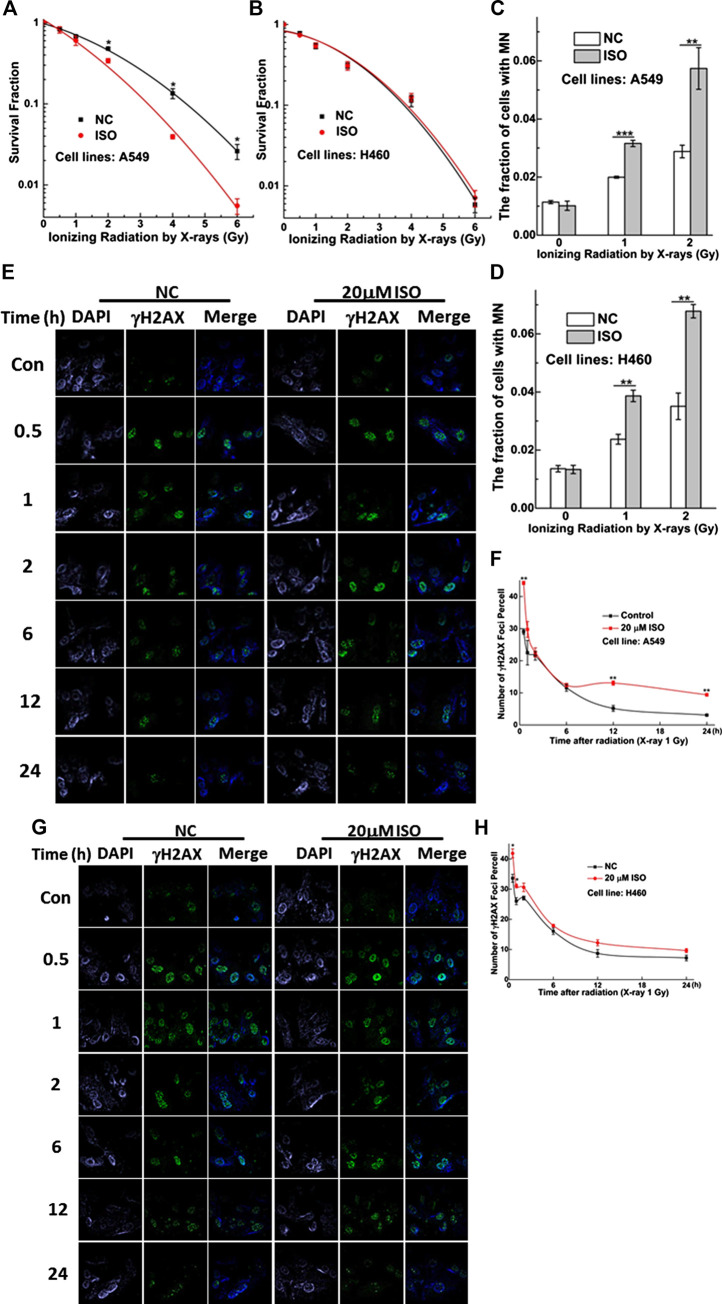
ISO sensitized NSCLC cells to IR. Survivals measured by colony formation assay in A549 **(A)** and H460 **(B)** cells pretreated with/without ISO and followed by 0, 0.5, 1, 2, 4, and 6 Gy X-rays irradiation. The fraction of MN in A549 **(C)** and H460 **(D)** cells pretreated with/without ISO and followed by 0, 1, and 2 Gy X-rays irradiation. Five hundred cells were scored under microscopy to determine the frequency of cell with micronuclei. Representative images of γH2AX foci (green) in A549 **(E)** and H460 **(G)** cells pretreated with/without 20 μM ISO for 24 h and then exposed to 1 Gy X-rays, fixed at different time points, and detected by immunofluorescence staining assay. The numbers of γH2AX foci in 100 cells of each group were counted at each time point in A549 **(F)** and H460 **(H)** cells. Each data point represents the mean of three separate experiments. *p < 0.05, **p < 0.01, ***p < 0.001 vs. non-drug-treated cells.

In A549 cells, treatment with ISO and irradiation decreased the viability (Figure 2A) and increased the MN fraction (Figure 2C) compared to the IR alone, especially at radiation doses of 2, 4, and 6 Gy. As shown in Figures 2E,F, treatment with ISO and irradiation significantly increased the numbers of γH2AX foci per cell, compared with IR alone at 1 Gy for 0.5 h (*p* < 0.01) in A549 cell lines. In addition, the dissolution of foci was faster in cells treated with ISO and irradiation from 0.5 to 6 h, compared to the IR alone. Interestingly, the number of γH2AX foci per cell in ISO + IR group was higher than that in the IR group from 12 to 24 h (Figure 2F).

As shown in Figure 2D, the MN fraction for H460 cells treated with ISO and irradiation was greater than that for cells treated with irradiation alone. However, this difference was not found by means of the colony formation assay (Figure 2B). As shown in Figures 2G,H, the number of γH2AX foci per cell in the ISO + IR group was higher than that in the IR group from 0.5 to 1 h after a radiation dose of 1 Gy (*p* < 0.01). However, this difference in the numbers of γH2AX foci per cell between ISO + IR and IR groups was not found in H460 cells after 2 h, indicating that ISO enhances the radiosensitivity of A549 cells and inhibits the repair of damaged DNA induced by irradiation.

### Treatment With Isorhamnetin Enhances Irradiation-Induced Cell Apoptosis and the Mitochondrial Membrane Potential

**FIGURE 3:**
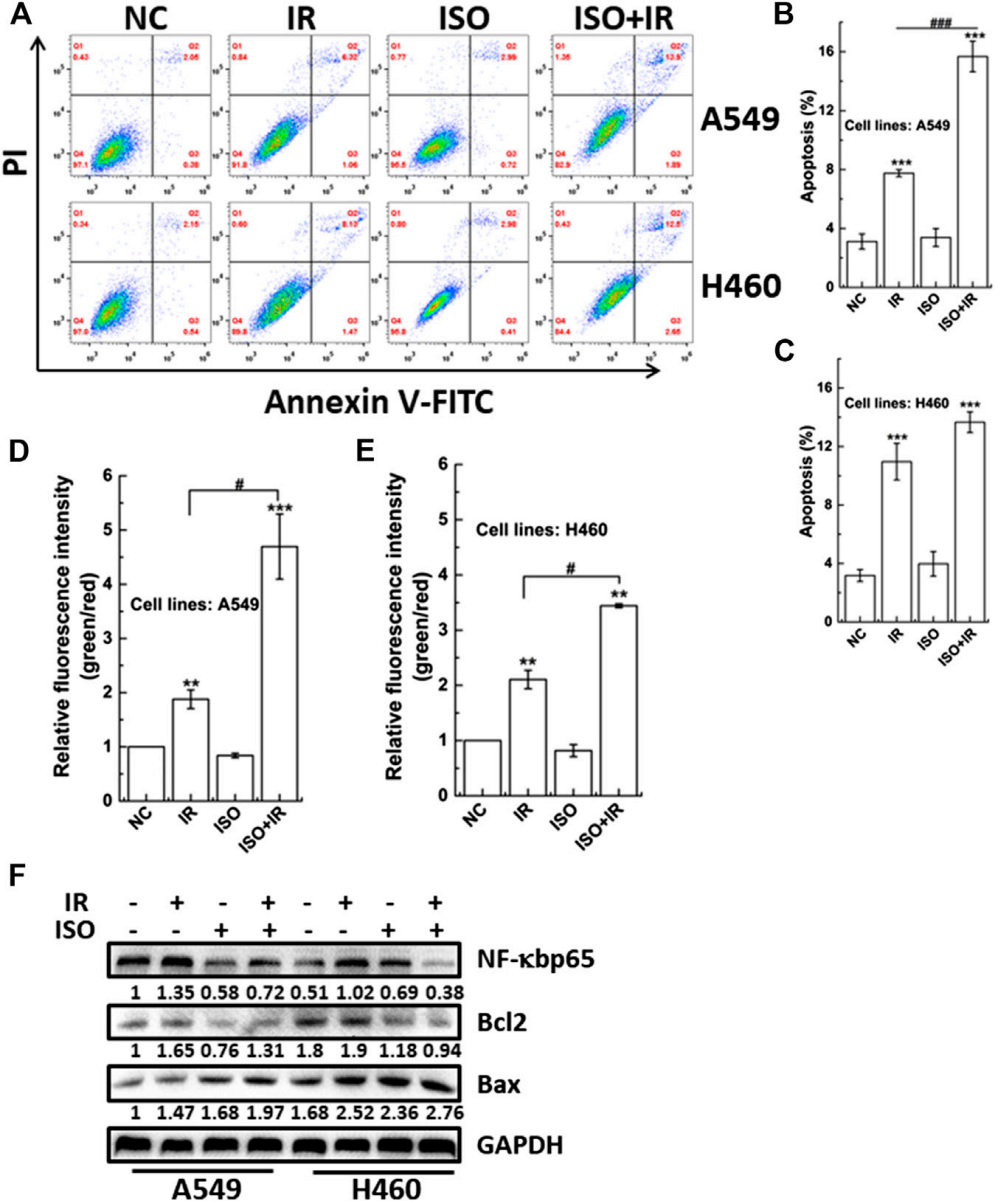
The impact of ISO combined irradiation on cell apoptosis and the MMP. The levels of apoptosis in A549 **(A**,**B)** and H460 **(A**,**C)** cells pretreated with/without ISO and then exposed to 2 Gy X-rays irradiation. The irradiated cells were sequentially cultured for 48 h and then harvested to be analyzed by an Annexin V/PI flow cytometry analysis. The MMP was quantified by the microplate reader in A549 **(D)** and H460 **(E)** cells pretreated with/without ISO and followed by 2 Gy X-rays at 24 h after irradiation. **(F)** The expression levels of apoptosis-related proteins were assessed and quantified by western blotting in A549 and H460 cells. Cells were pretreated with/without ISO for 24 h and followed by 0 or 2 Gy X-rays and then analyzed by western blot after 12 h. ***p < 0.001 vs. the control group. ^###^ *p* < 0.001 vs. non-drug-treated cells.

Apoptosis is one type of programmed cell death. We investigated the apoptosis rate in A549 and H460 cells treated with ISO and irradiation or irradiation alone using flow cytometry (Figure 3A). Figure 3B shows that the apoptosis rate was higher in A549 cells treated with ISO and irradiation than that in cells treated with irradiation alone. However, this difference in the apoptosis rate between ISO + IR and IR groups was not found in H460 cells (Figure 3C).

The MMP is a key indicator of mitochondrial function and activity, and mitochondrial depolarization has been reported to associate with apoptosis. Mitochondrial depolarization is indicated by decreased red fluorescence and increased green fluorescence after JC-1 staining, and the collapse of the MMP is revealed by the green/red fluorescence intensity ratio. We investigated whether the MMP was affected in cells treated with ISO and irradiation. Figures 3D,E show that ISO treatment significantly enhanced the radiation-induced collapse of MMP (increase in the green/red fluorescence intensity ratio) in both A549 and H460 cells.

We also examined the expression of proteins associated with apoptosis. As shown in Figure 3F, ISO treatment significantly suppressed the upregulation of radiation-induced NF-κBp65 expression in A549 and H460 cells. In addition, the expression of NF-κBp65 in A549 cells was higher than in H460 cells, which may be associated with the difference of radiosensitivity among different cell lines pretreated by ISO. Interestingly, ISO treatment decreased the Bcl2 level and increased the Bax level, compared to the irradiation alone (Figure 3F), indicating that ISO and irradiation accelerated the decrease of the of Bcl2/Bax ratio. Taken collectively, these results indicated that ISO treatment facilitated the apoptosis after irradiation.

The radiosensitization of ISO in H460 cells was not significant, reflected by clonogenic survival and apoptosis endpoints. Therefore, the mechanism of ISO radiosensitivity was widely investigated only in A549 cells in the following experiments.

### Treatment With Isorhamnetin Upregulates the Irradiation-Induced Interleukin-13 Level

**FIGURE 4:**
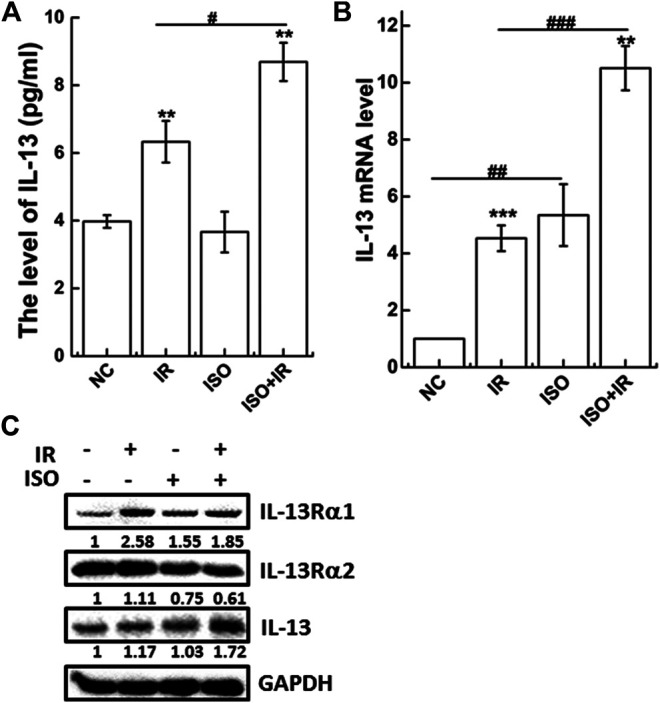
ISO enhances the levels of anti-inflammatory IL-13 activated by IR in A549 cells. The level of IL-13 **(A)** in supernatant of cells pretreated with/without ISO for 24 h, exposed to 2 Gy X-rays, and then detected 12 h after irradiation by MSD assay. The expression of IL-13 gene **(B)** and associated receptor proteins **(C)** were analyzed at 12 h after irradiation by qRT-PCR and western blot, respectively, in A549 cells pretreated with/without ISO for 24 h and then exposed to 2 Gy X-rays. Fold changes over negative control were presented from 2^−ΔΔCt^ formula. *p < 0.05, **p < 0.01, ***p < 0.001 vs. the control group. ^#^ *p* < 0.05, ^##^ *p* < 0.01, and ^###^ *p* < 0.001 vs. non-drug-treated cells.

To widely identify immune-related protein involved in the radiosensitization of ISO in A549 cells, the levels of 10 cytokines (IL-6, IL-8, IL-10, IL-13, IL-1β, IL-2, IL-4, IFN-γ, IL-12p70, and TNF-α) were measured using the Meso Scale Discovery (MSD) platform. MSD is a highly sensitive high-throughput electrochemiluminescence measurement system, which can detect as little as ∼0.29 pg/ml of a target cytokine (Osuchowski et al., 2005). We found that the IL-13 level in the ISO + IR group (8.6886 ± 0.5642 pg/ml) was higher than that in the IR group (6.3332 ± 0.6143 pg/ml). The levels of IL-13 expression in negative control and ISO treatment groups were 3.9742 ± 0.1879 pg/ml and 3.6637 ± 0.6046 pg/ml, respectively (Figure 4A).

IL-13 can suppress the production of the inflammatory cytokine TNF and inhibit NF-κB activation by preventing degradation of IκBα (Lentsch et al., 1997; Manna and Aggarwal, 1998). A previous study has demonstrated that IL-13 is a mediator, and possibly a therapeutic target, in radiation-induced lung injury, as shown by saturating fraction of the circulating decoy receptor IL-13Rα2 (Chung et al., 2016). Therefore, ISO may upregulate the expression of IL-13, thereby inhibiting NF-κB activation, inducing apoptosis, and triggering radiosensitivity.

IL-13 mRNA and protein expression were analyzed by qRT-PCR (Figure 4B) and western blot (Figure 4C), respectively. As shown in Figure 4C, ISO treatment upregulated the IL-13 mRNA levels compared to the negative control. Treatment with ISO and irradiation also significantly increased IL-13 mRNA and protein levels compared to irradiation alone. Furthermore, the expression of IL-13Rα2, the high affinity binding decoy receptor for IL-13, was significantly decreased in the ISO + IR group compared to the IR group (Figure 4C), indicating that ISO sensitizes cells to irradiation by affecting the function of IL-13.

### Interleukin-13 Is Required for the Radiosensitivity Mediated by Isorhamnetin

**FIGURE 5:**
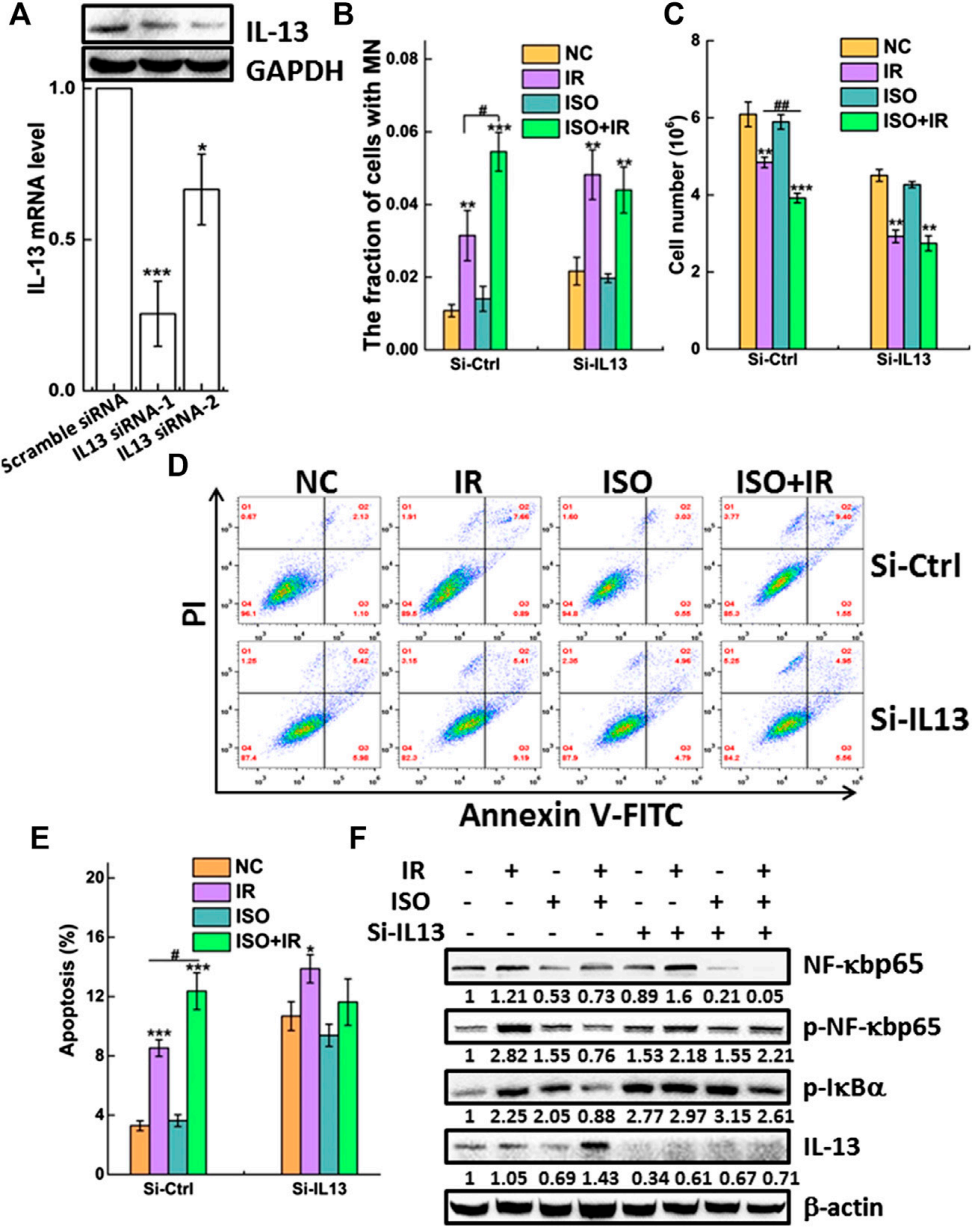
IL-13 is required for the radiosensitivity mediated by ISO. **(A)** A549 cells were transiently transfected with IL-13 siRNA or scramble-siRNA as a control. Forty-eight hours after transfection the knockdown efficiency of IL-13 in cells was confirmed by western blot and qRT-PCR assays. Knockdown of IL-13 disturbed radiosensitivity mediated by ISO indicated in si-Ctrl or si-IL13 groups by micronucleus **(B)**, cell growth **(C),** and apoptosis assays **(D**,**E)**. **(F)** The expression of phosphorylated and total NF-κBp65 and p-IκBα, as well as IL-13 and β-actin, was assessed in negative vector or IL-13 silenced A549 cells by western blotting, respectively.

To confirm the effects of ISO on the radiosensitivity of cells through the increased expression of IL-13, we silenced IL-13 by RNA interference and measured several relevant biological endpoints. The most effective IL-13 siRNA, as confirmed by qRT-PCR and western blot (Figure 5A), was selected for further studies. As shown in Figure 5B, IL-13 knockdown (si-IL13 cells) failed to increase MN formation in ISO + IR group, compared to the IR group. When the cells were pretreated with 20 μM ISO and then irradiated with a radiation dose of 2 Gy, the cell number decreased to 80.9 ± 3.2% (green bar) compared with irradiation alone (set as 100%, purple bar) (Figure 5C). However, there was no significant toxicity in si-IL13 A549 cells after ISO treatment and irradiation (Figure 5C).

We measured the apoptosis rate in si-IL13 cells after ISO treatment and irradiation. Figures 5D,E show that irradiation did not increase the apoptosis rate in si-IL13 cells treated with ISO, compared to cells without ISO treatment. However, the apoptosis rate in IL-13 silenced control cells was higher than that in mock silenced cells (si-ctrl), which may be associated with the anti-inflammatory role of IL-13.

Figure 5F shows that ISO treatment significantly increased IR-induced IL-13 expression and inhibited the IR-induced activation of NF-κB by decreasing p-NF-κBp65 and the p-IκBα expression, compared to the IR group in si-ctrl group. Intriguingly, we observed that IL-13 knockdown also promoted the expression of p-IκBα and p-NF-κBp65 in control group, allowing for its nuclear translocation and regulation of gene expression. However, the upregulation of p-NF-κBp65 induced by IR was not suppressed by ISO in si-IL13 cells, suggesting that the gene of IL-13 pathway is required in the activation of NF-κB agitated by IR. Together, these results indicated that ISO treatment further increased the radiation-mediated upregulation of the IL-13 level, which inhibits the activation of NF-κB, thereby promoting apoptosis and enhancing radiosensitivity.

## Discussion

Radiotherapy is commonly used to treat cancer, with more than 50% of cancer patients receiving radiotherapy during the clinical management of the disease. Unfortunately, the 5-year survival rate of radiotherapy alone is only 5–10% in lung cancer patients (Mauguen et al., 2012). The main cause of the poor response is the intrinsic or acquired resistance of lung cancer to radiotherapy (You et al., 2014). Radiosensitizers, sensitizing cancer cells to the effects of irradiation but protecting normal cells from its deleterious effects, play an important role in radiotherapy. For example, ISO (a 3′-O-methylated metabolite of quercetin) has been studied for its anticancer and antioxidant activity and anti-inflammatory properties, as well as its ability to induce chemosensitivity in several human cancer cells (Ramachandran et al., 2012; Saud et al., 2013; Yang et al., 2013; Li et al., 2014; Ahn and Lee, 2017). Due to its lower toxicity, ease of oral administration, and affordability, we investigated its mechanism of radiosensitization in order to provide a foundation for its development as a naturally occurring radiosensitizer of cancer cells. We selected A549 (lung adenocarcinoma) and H460 (large lung cell carcinoma) cell lines as representative models of NSCLC (Ji et al., 2018). We found that treatment with ISO, followed by irradiation, significantly decreases the surviving fraction in A549 cells and increases the MN fraction in A549 and H460 cells. In addition, we found that treatment with ISO + IR could significantly enhance DNA damage, the collapse of MMP, and the apoptotic rate, suggesting that ISO treatment can significantly enhance the radiosensitivity of A549 cells. We did not find the significant decrease of the survival and the increase of apoptosis in H460 cells after ISO pretreatment followed by IR. It may be because of the different biological endpoints having different sensitivity at certain timepoint after stress (Hu et al., 2005). In addition, this difference may be caused by the low expression of NF-κB in H460 cells, which weaken the role of ISO radiosensitivity. In A549 cells, NF-κB expression is high, the activation of NF-κB has a greater effect, and ISO treatment enhances more effective radiosensitivity.

Prior studies have indicated that ISO exerts its anti-inflammatory effects through the deactivation of NF-κB (Zhang et al., 2011; Yang et al., 2013). The activation of the NF-κB signaling pathway confers lung cancer cell radioresistance and thus survival by interfering with apoptotic signals. For example, c-IAP1 and c-IAP2 either directly block caspase function or indirectly induce ubiquitination in irradiated cancer cells (Varfolomeev et al., 2008). Furthermore, irradiation can induce DNA damage, which evokes a response where NF-κB is activated to protect cells from inflammation and subsequent death. Our results demonstrated that ISO treatment suppressed the irradiation-mediated activation of the NF-κB signaling pathway, which promoted programmed cell death.

IL-13, a pleiotropic immune regulatory cytokine, exhibits both immunomodulatory and anti-inflammatory properties. The anti-inflammatory effects of IL-13 are supported by its ability to downregulate the level of lipopolysaccharide-induced macrophage inflammatory protein-1α and the production of proinflammatory cytokines, namely, IL-1, TNF, IL-6, IL-8, IL-10, and IL-12 in monocytes (Yang et al., 2013). In addition, blocking the IL-13 mediated phosphorylation of STAT6 can protect breast cancer cells from developing sensitivity to irradiation (Rahal et al., 2018). In particular, IL-13 is a prominent feature in causing barrier effects and the epithelial apoptosis in cell models (Heller et al., 2005). Consistent with our findings in NSCLC cells, ISO treatment and irradiation increased the IL-13 level, which induced apoptosis of si-ctrl cells by downregulating p-IκBα and inhibiting NF-κB activation (p-NF-κBp65). IL-13 not only acts an apoptotic effector but also has a profound effect. We also found that the IR-induced NF-κB signaling activation was inhibited by ISO treatment, and it was abrogated in IL-13 silenced cells, which implied that NF-κB activation is inhibited by IL-13. Other studies have demonstrated IL-4 and IL-13 bind to the same receptors and exert similar biological functions by inhibiting NF-κB dependent transcription (Bennett et al., 1997; Shinozaki et al., 2010). In this study, the IL-4 expression was also upregulated in A549 cells after X-rays irradiation but not enhanced after treatment with ISO (data not shown). Transcriptional regulatory mechanisms are complex, and further studies are needed to completely understand the molecular mechanisms responsible for the ISO-mediated increase in IL-13 expression and decrease in NF-κB activation after radiation.

Studies have proven that autophagy has been revealed as a novel response of cancer cells to ionizing radiation (Klionsky and Emr, 2000; Djavaheri-Mergny et al., 2006; Xiao, 2007). In our study, ISO treatment induces the formation of vacuolation in A549 cells, which may be involved in autophagic cell death. Therefore, we speculated the occurrence of mitochondrial dysfunction after ISO treatment and irradiation, resulting in autophagy in A549 cells. It is, therefore, necessary to further investigate the mechanism involved in sensitizing effect of ISO.

**FIGURE 6:**
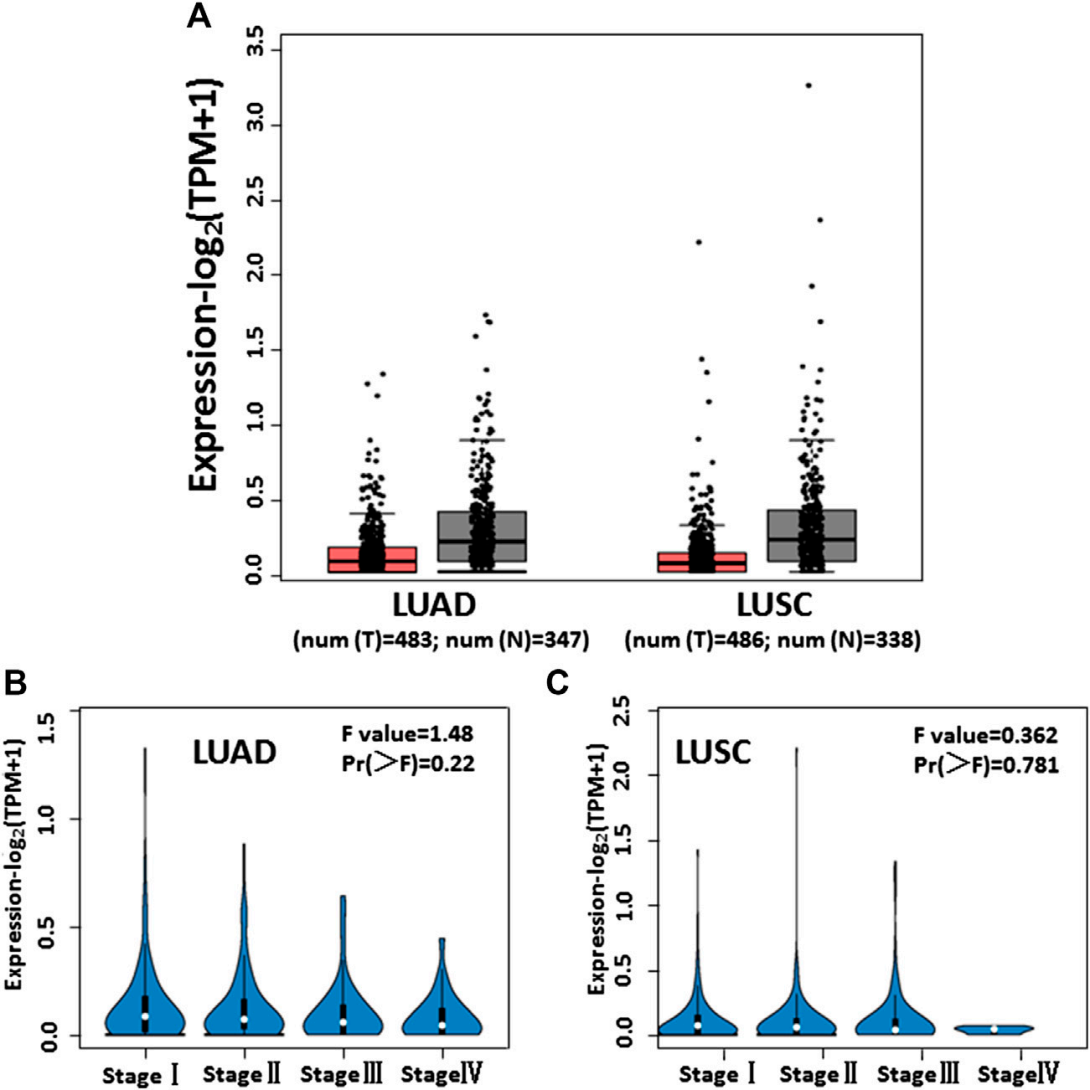
The levels of IL-13 expression in NSCLC cells analyzed according to cancer public database. **(A)** IL-13 expression profile across tumor samples and adjacent normal tissues from GEPIA in LUAD and LUSC. **(B**,**C)** IL-13 expression levels in different stages of LUAD and LUSC from GEPIA database.

The GEPIA database (http://gepia.cancer-pku.cn/index.html) is a comprehensive web-based analysis tool that provides information on the expression of different genes in tumor and normal tissue specimens. We browsed the GEPIA database and observed low IL-13 expression in 483 LUAD and 486 LUSC tumor specimens, compared with 347 LUAD and 338 LUSC nontumor specimens (Figure 6A). From stage I to stage IV, the expression of IL-13 gradually decreased in LUAD (Figure 6B) and LUSC (Figure 6C) specimens, indicating that the changes in the IL-13 level can serve in evaluation of NSCLC. These results also suggested that IL-13 may be a biomarker of the disease and a therapeutic target in NSCLC.

## Conclusion

To our best knowledge, this is the first study to report that ISO treatment enhanced the radiosensitivity of lung cancer cells through IL-13 and the NF-κB signaling pathway, thereby promoting programmed cell death. These findings provided new insights into the mechanism responsible for the effects of ISO and may guide the development of novel therapeutic approaches for lung cancer.

## Data Availability Statement

The raw data supporting the conclusions of this article will be made available by the authors, without undue reservation.

## Author Contributions

YD designed and performed experiments, produced most data, analyzed all results, and wrote the manuscript. CJ and YaL were involved in the production and analysis of clonogenic assays and MN formation assays. JW and YeL reviewed and edited the manuscript. KS directed the study, provided most resources and funding, and reviewed the manuscript. All authors critically read and approved the final content of the manuscript.

## Funding

This study was supported by the National Natural Science Foundation of China (11705248, 31660060) and the Science and Technology Research Project of Gansu Province (No. 145RTSA012 and 17JR5RA307).

## Conflict of Interest

The authors declare that the research was conducted in the absence of any commercial or financial relationships that could be construed as a potential conflict of interest.
